# When the Phagosome Gets Leaky: Pore-Forming Toxin-Induced Non-Canonical Autophagy (PINCA)

**DOI:** 10.3389/fcimb.2022.834321

**Published:** 2022-03-17

**Authors:** Marc Herb, Alexander Gluschko, Alina Farid, Martin Krönke

**Affiliations:** ^1^ Faculty of Medicine and University Hospital of Cologne, Institute for Medical Microbiology, Immunology and Hygiene, Cologne, Germany; ^2^ Cologne Cluster of Excellence in Cellular Stress Responses in Aging-Associated Diseases (CECAD), University of Cologne, Cologne, Germany; ^3^ German Center for Infection Research, Bonn-Cologne, Germany

**Keywords:** non-canonical autophagy, macrophages, ULK complex, pore-forming toxins, macroautophagy, xenophagy, LC3-associated phagocytosis, PINCA

## Abstract

Macrophages remove bacteria from the extracellular milieu *via* phagocytosis. While most of the engulfed bacteria are degraded in the antimicrobial environment of the phagolysosome, several bacterial pathogens have evolved virulence factors, which evade degradation or allow escape into the cytosol. To counter this situation, macrophages activate LC3-associated phagocytosis (LAP), a highly bactericidal non-canonical autophagy pathway, which destroys the bacterial pathogens in so called LAPosomes. Moreover, macrophages can also target intracellular bacteria by pore-forming toxin-induced non-canonical autophagy (PINCA), a recently described non-canonical autophagy pathway, which is activated by phagosomal damage induced by bacteria-derived pore-forming toxins. Similar to LAP, PINCA involves LC3 recruitment to the bacteria-containing phagosome independently of the ULK complex, but in contrast to LAP, this process does not require ROS production by Nox2. As last resort of autophagic targeting, macrophages activate xenophagy, a selective form of macroautophagy, to recapture bacteria, which evaded successful targeting by LAP or PINCA through rupture of the phagosome. However, xenophagy can also be hijacked by bacterial pathogens for their benefit or can be completely inhibited resulting in intracellular growth of the bacterial pathogen. In this perspective, we discuss the molecular differences and similarities between LAP, PINCA and xenophagy in macrophages during bacterial infections.

## Phagocytosis: Main Weapon of Macrophages

The most prominent, characteristic feature of macrophages is their ability to phagocytose extracellular material ranging from cellular debris to whole cells (Fadok et al., 1998; Erwig and Henson, 2008; Kono and Rock, 2008; Suzanne and Steller, 2013; Kourtzelis et al., 2020), but also invading pathogens (Djaldetti et al., 2002; Haas, 2007; Lemke, 2019). With this ability to separate foreign invaders like bacteria, fungi or parasites from the rest of the organism, they represent one of the first lines of defense against invading pathogens (Rosales and Uribe-Querol, 2017). After induction by various cell surface receptors, such as the mannose receptor, Fc‐receptors and scavenger receptors (Freeman and Grinstein, 2014; Uribe-Querol and Rosales, 2020), the cargo is enclosed in a single-membrane structure called phagosome. Several factors, like vacuolar-type H^+^-ATPase (V-ATPase)-mediated acidification (Sun-Wada et al., 2009; Dragotakes et al., 2020; Westman and Grinstein, 2021), production of reactive oxygen species (ROS) (Craig and Slauch, 2009; Slauch, 2011; Wink et al., 2011; Herb and Schramm, 2021) and exposure to hydrolases after fusion with lysosomes (del Cerro-Vadillo et al., 2006; Schramm et al., 2014; Weiss and Schaible, 2015) lead to the formation of a highly antimicrobial environment for engulfed pathogens and, in most cases, result in their degradation (Haas, 2007). However, several bacterial pathogens have established strategies to evade this degradative fate in the phagosome (Mitchell et al., 2016; Grijmans et al., 2022), e.g. *Staphylococcus aureus* (*S. aureus*) (Fraunholz and Sinha, 2012; Moldovan and Fraunholz, 2019; Rao et al., 2020), *Salmonella typhimurium* (*S. typhimurium*) (Eriksson et al., 2003; Fenlon and Slauch, 2014; Burton et al., 2014; Rhen, 2019; Rao et al., 2020) or *Mycobacterium tuberculosis* (Queval et al., 2017; Koster et al., 2017), which can alter the phagosomal composition and structure for their benefit and do not only remain unharmed, but also replicate inside the phagosome.

## LC3-Associated Phagocytosis

**Figure 1:**
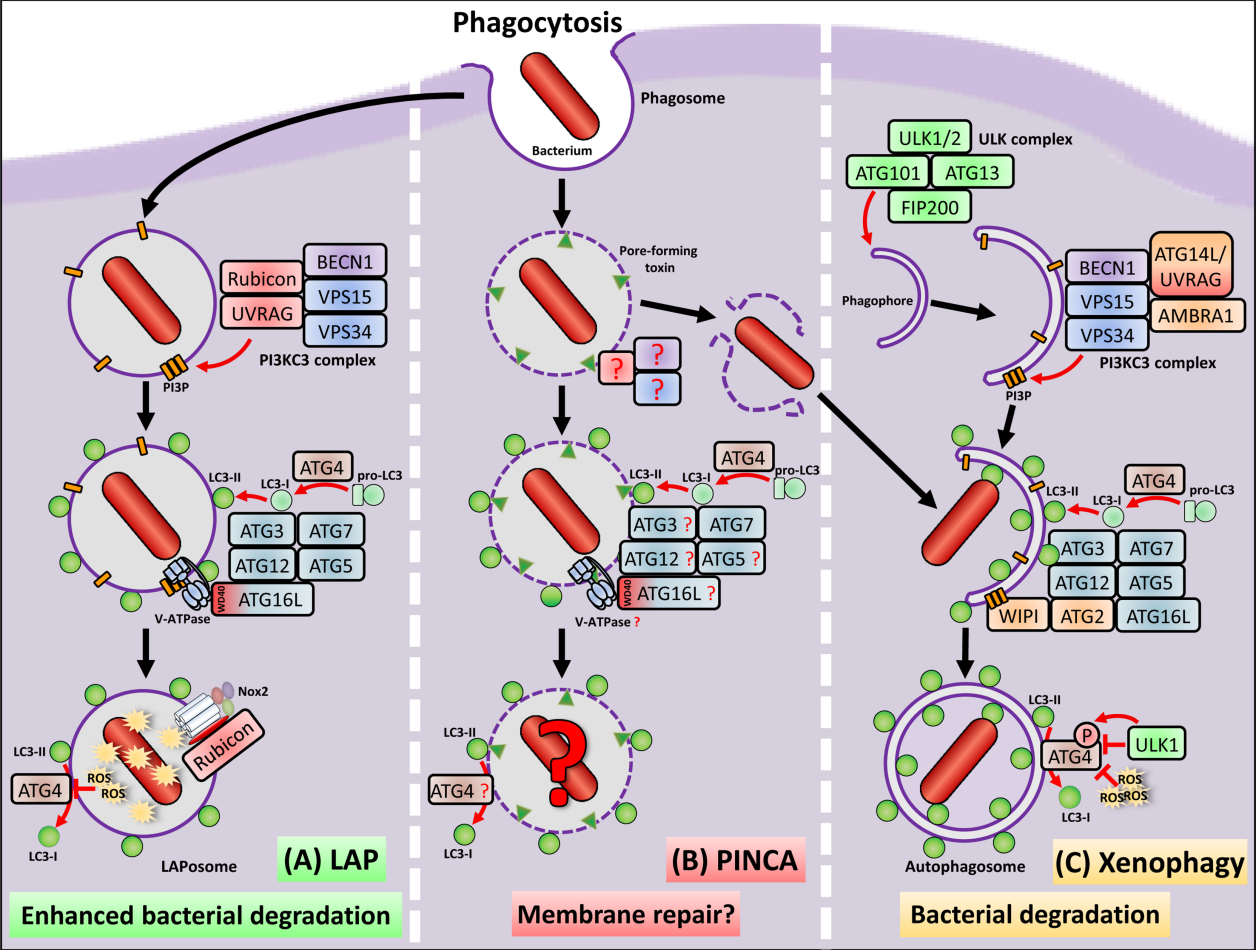
Macrophages remove bacteria from the extracellular milieu via phagocytosis, which is the engulfment and enclosure in a single membrane vesicle, called phagosome. Most of the engulfed bacteria are degraded due to the highly antimicrobial environment of the phagosome. However, several bacterial pathogens have evolved virulence factors, which allow escape from the phagosome into the cytosol. **(A)** Macrophages activate LAP to inactivate and destroy the bacterial pathogen before phagosomal escape. LAP shares some, but not all components of the autophagy machinery to conjugate LC3 to the phagosomal membrane. Importantly, LAP is completely independent from the ULK complex, defining it as non-canonical autophagy pathway. The PI3KC3 complex, which is activated during LAP, shares the core components BECN1, VSP15 and VSP34 but exclusively contains UVRAG and Rubicon, which is not only important for recruitment of the PI3KC3 complex, but also stabilizes the p22^phox^ subunit of Nox2. To the PI3P platforms formed by the PI3KC3 complex, a so far unknown factor binds to which ATG16L1 is recruited via its WD40 domain together with the LC3 conjugation machinery consisting of ATG3, ATG7, ATG5 and ATG12. A preprint study (Hooper et al., 2021) marks the V-ATPase as possible candidate, which recruits ATG16L1 and the LC3 conjugation machinery to the phagosome during LAP. ROS production by Nox2 leads to oxidative inactivation of the protease ATG4, which prevents ATG4-mediated deconjugation of LC3 and stabilization of the LAPosome. LAPosomes show enhanced fusion with lysosomes and enhanced degradation of the bacterial pathogen. **(B)** During PINCA, damage induced by bacteria-derived pore-forming toxins induces LC3 recruitment to the phagosome independent of the ULK complex components FIP200 and ULK1/2. With the exception of ATG7, the factors for induction and execution, which are necessary for LC3 recruitment during PINCA, as well as its precise functions remain to be resolved. Since pore-forming toxins lead to V-ATPase and ATG16L recruitment during other non-canonical pathways, this might also be the case during PINCA. **(C)** To recapture bacterial pathogens that had managed to escape from phagosomes, either during conventional phagocytosis, LAP or PINCA, macrophages activate xenophagy, a selective form of macroautophagy. Initiation of xenophagy depends on the ULK complex, i.e. ULK1/2, FIP200, ATG13 and ATG101, which generates a double-membrane structure called phagophore. Recruitment of the proteins BECN1, VSP15, VSP34, AMBRA1 and either UVRAG or ATG14L to the phagophore leads to formation of the PI3KC3 complex, which then generates PI3P in the growing phagophore membrane. These PI3P platforms serve as docking station for the WIPI-ATG2 complex, which subsequently recruits the machinery for LC3 conjugation, facilitating degradation of the recaptured bacterial pathogen in autophagolysosomes. During xenophagy, inactivation of ATG4 can be mediated by cytotosolic ROS production or phosphorylation of ATG4 by ULK1 both preventing deconjugation of LC3 from the autophagosome.

Since several bacterial pathogens can evade the degradative fate of the phagosome, macrophages activate a non-canonical autophagy pathway called LC3-associated phagocytosis (LAP), which can enhance phagolysosomal fusion. For example, phagosomes containing Toll-like receptor ligand-coated latex beads (Sanjuan et al., 2007), dead cells (Martinez et al., 2011) or pathogens, such as *Legionella dumoffii* (Hubber et al., 2017), *Listeria monocytogenes (L. monocytogenes)* (Gluschko et al., 2018) and *Aspergillus fumigatus* (Martinez et al., 2015) show increased fusion with lysosomes during LAP, resulting in enhanced degradation of the cargo. Notably, LAP can also delay phagolysosomal fusion, leading to prolonged antigen presentation by major histocompatibility complex (MHC) class II (Romao et al., 2013; Ma et al., 2014; Fletcher et al., 2018). LAP is induced by various surface receptors found on macrophages (Sanjuan et al., 2007; Ma et al., 2012; Tam et al., 2014; Gluschko et al., 2018; Hayashi et al., 2018) and results in the decoration of phagosomes with microtubule-associated protein 1 light chain-3 (LC3) family proteins, resulting in so called LAPosomes. (Sanjuan et al., 2007; Martinez et al., 2011; Durgan et al., 2021). LAP and macroautophagy share some but not all components of the autophagic machinery, e.g. parts of the class III phosphatidylinositol 3-kinase (PI3KC3) complex or the two ubiquitin-like conjugation systems, i.e. the autophagy-related protein (ATG) 12 conjugation system and the LC3 lipidation system (Florey et al., 2011; Lystad et al., 2019) (**Figure 1**). Generation of LAPosomes requires the production of the membrane lipid phosphatidylinositol 3-phosphate (PI3P) (by components of the PI3KC3 complex) and ATG16L1 recruitment to the PI3P-containing target membrane (Kim et al., 2013; Martinez et al., 2015). Comparable to macroautophagy, the PI3KC3 complex, which is involved in LAPosome formation, also contains Beclin-1 (BECN1) (Sanjuan et al., 2007; Martinez et al., 2015; Backer, 2016), vacuolar protein sorting-associated proteins (VPS) 15 and 34, as well as UV radiation resistance-associated gene protein (UVRAG), but lacks ATG14 and activating molecule in BECN1-regulated autophagy protein 1 (AMBRA1) (Martinez et al., 2015; Backer, 2016). In contrast to macroautophagy, LAP requires a specific component of the PI3KC3 complex called Rubicon, which facilitates VPS34 activity and sustains PI3P presence on the LAPosome (Martinez et al., 2015). Another difference is the dispensability of WD repeat domain phosphoinositide-interacting proteins (WIPI) and ATG2, which are required for macroautophagy but not for LAP (Martinez et al., 2015; Fischer et al., 2020). Recently, it was shown that the WD40 domain of ATG16L1 is required for its recruitment to the PI3P-containing target membrane during LAP. Mice lacking the WD40 domain of ATG16L1 are deficient for LAP but not for macroautophagy (Rai et al., 2019). This implicates that a complete different factor than WIPI is required for recruitment of ATG16L1 to the PI3P-containing membrane on the LAPosome. Some studies have shown that the V-ATPase can recruit ATG16L1 onto single-membrane vesicles *via* its WD40 domain (Florey et al., 2015; Fletcher et al., 2018; Xu et al., 2019; Fischer et al., 2020). Moreover, activity of V-ATPase can be induced by osmotic imbalances caused by pore-forming toxins e.g. by the *Helicobacter pylori (H. pylori)* pore-forming toxin vacuolating cytotoxin A (VacA) (Florey et al., 2015). V-ATPase activation and ATG16L1 recruitment lead to LC3 lipidation onto single-membrane vacuoles, a mechanism which is independent of the upstream macroautophagy machinery, e.g. the unc-51-like kinase (ULK) complex (Fletcher et al., 2018; Xu et al., 2019), which resembles LAP. A recent preprint study by Hooper et al. indicates that also during LAP, V-ATPase is responsible for ATG16L1 recruitment and subsequent conjugation of LC3 onto the phagosomal membrane (Hooper et al., 2021) (**Figure 1**). LC3 lipidation resembles the conjugation of ubiquitin to proteins (Slobodkin and Elazar, 2013), therefore similar terms were used for the enzymes of the two ubiquitin like conjugation systems, which carry out the process (Ichimura et al., 2000). For both complexes E1-like ATG7 and E2-like ATG3 catalyze the reactions during LC3 lipidation. The ATG12–ATG5–ATG16L1 complex carries out an analogous function as the ubiquitin E3 ligase and mediates transfer of LC3 from ATG3 to phosphatidylethanolamine (PE) in the phagosomal membrane (Hanada et al., 2007; Sakoh-Nakatogawa et al., 2013).

Notably, Durgan and colleagues have shown that single-membrane structures during non-canonical autophagy, e.g. LAP, show a different LC3 lipidation pattern. In addition to PE-conjugated LC3, the study demonstrated that LC3 is also conjugated to phosphatidylserine (PS). This is in contrast to autophagosomes during macroautophagy, which exclusively display PE-conjugated LC3 (Durgan et al., 2021). However, the major difference beyond LC3 conjugation onto single- vs. double-membrane vesicles is that LAP, in contrast to macroautophagy, is completely independent of the ULK complex (Martinez et al., 2011; Martinez et al., 2015). Most importantly, LC3 recruitment to phagosomes by LAP requires production of ROS by the NADPH oxidase 2 (Nox2) (Huang et al., 2009; Yang et al., 2012; Martinez et al., 2015; Gluschko et al., 2018; Gluschko et al., 2021; Herb and Schramm, 2021; Ligeon et al., 2021). The Nox2 complex consists of two integral membrane subunits, gp91^phox^, and p22^phox^, and the cytosolic subunits p67^phox^, p47^phox^, p40^phox^ and Rac1/2 (Herb and Schramm, 2021). For induction of ROS production by Nox2, the cytosolic subunits are recruited to the integral membrane subunits. Thereby, Rubicon plays an important role beyond its involvement in generation of PI3P by the PI3KC3 complex, by stabilizing p22^phox^ *via* direct binding (Yang et al., 2012; Martinez et al., 2015). However, why Nox2-derived ROS are crucial for LAP induction is not understood in detail. Notably, it was recently shown that ROS production by Nox2 mediates oxidative inactivation of the protease ATG4 thereby preventing deconjugation of LC3 from the phagosome (Ligeon et al., 2021).

## PINCA – a New Non-Canonical Autophagy Pathway

We recently described a new variant of non-canonical autophagy in macrophages, which we termed pore-forming toxin-induced non-canonical autophagy (PINCA) (Gluschko et al., 2021). Similar to other forms of non-canonical autophagy, e.g. LAP, PINCA also is independent of the ULK complex components focal adhesion kinase family interacting protein of 200 kD (FIP200) and ULK1/2. Importantly, in contrast to LAP, PINCA did also not require Nox2-derived ROS production, which is crucial for LC3 decoration of phagosomes during LAP (Sanjuan et al., 2007; Martinez et al., 2015; Gluschko et al., 2018; Herb et al., 2020; Ligeon et al., 2021). Thus, we observed induction of PINCA in Nox2-deficient peritoneal macrophages (PM) and in wildtype bone marrow-derived macrophages (BMDM), which fail to produce sufficient levels of ROS for induction of LAP due to low expression of Nox2 (Gluschko et al., 2021). Instead, damage induced by the pore-forming toxin listeriolysin O (LLO) of *L. monocytogenes* or by the several pore-forming toxins of *S. aureus* were necessary to induce LC3 recruitment to the damaged phagosomes, i.e. PINCA (**Figure 1**).

Notably, damage induced by the needle-like Type three secretion system (T3SS) of *Shigella flexneri (S. flexneri) or S. typhimurium* did not induce PINCA in macrophages. A possible reason for this is the expression of bacterial virulence factors such as Salmonella outer protein F (SopF) of *S. typhimurium*, which inhibits the vacuolar V-ATPase and thereby prevents ATG16L recruitment and LC3 lipidation onto *S. typhimurium*-containing vacuoles (Xu et al., 2019). Interestingly, vacuolar damage caused by SopF-deficient *S. typhimurium* triggered ATG16L recruitment and LC3 lipidation onto *S. typhimurium*-containing vacuoles in epithelial cells (Xu et al., 2019), which was independent of FIP200 and resembled PINCA. Thus, it is likely that also in macrophages T3SS-induced damage can trigger PINCA, when this process is not actively inhibited by bacterial virulence factors such as SopF. Notably, this T3SS-induced LC3 lipidation on damaged, but still intact vacuoles/phagosomes should not be mistaken with xenophagy, a selective form of macroautophagy, which targets ruptured vacuoles, membrane remnants or cytosolic bacteria and involves exposure of glycans and the recruitment of galectins and several other factors such as TANK-binding kinase 1 (TBK1) (Thurston et al., 2012; Ravenhill et al., 2019; Bell et al., 2021). As already mentioned, in mouse embryonic fibroblasts (MEFs) pore-forming toxins can induce osmotic imbalances within endolysosomal compartments, which are sensed by V-ATPase and result in ATG16L recruitment and LC3 conjugation (Florey et al., 2015). Due to this, it is plausible that bacterial toxin-induced pore formation during PINCA can also induce osmotic imbalances within phagosomes in macrophages. Wether these osmotic imbalances can trigger V-ATPase-coupled ATG16L recruitment and LC3 lipidation during PINCA, as observed during other non-canonical autophagy pathways (Florey et al., 2015; Fletcher et al., 2018; Xu et al., 2019; Hooper et al., 2021), are interesting topics for future studies.

The functional purpose of PINCA seems to be related to LAP during *L. monocytogenes* infection of wildytpe PM. We observed that during PINCA, LC3-positive phagosomes fused more often with lysosomes than conventional, LC3-negative phagosomes indicating that also PINCA promotes phagolysosomal fusion (Gluschko et al., 2021). However, in sharp contrast to LAP, which clearly promotes the anti-listerial activity of tissue macrophages, e.g. PM (Gluschko et al., 2018), LC3 recruitment to phagosomal membranes by PINCA and subsequently increased phagolysosomal fusion did not substantially contribute to anti-listerial activity of BMDM (Gluschko et al., 2021). Both LAP and PINCA require ATG7 for LC3 conjugation (Herb et al., 2020). Therefore, the question whether PINCA contributes to anti-listerial activity could unfortunately not be answered by the sole use of ATG7-deficient macrophages (Gluschko et al., 2021), since these cells can induce neither LAP nor PINCA. In addition, Nox2-deficient PM are not well suited to investigate the functional purpose of PINCA, since Nox2-derived ROS are not only necessary for LAP induction but also fulfill a plethora of other antimicrobial functions (Canton et al., 2021; Herb and Schramm, 2021). Moreover, ROS have been shown to inactivate ATG4, thereby preventing deconjugation of LC3 from the phagosome (Ligeon et al., 2021). It is conceivable that during PINCA, LC3 is continuously deconjugated from the phagosome in the absence of ROS production, except there is a, yet unknown, ROS-independent mechanism, which inactivates ATG4 during PINCA. Due to this, a possible antimicrobial function of PINCA could be easily overlooked, when PINCA is induced in the absence of ROS, which not only prevent LC3-deconjugation (Ligeon et al., 2021), but also substantially contribute to other antimicrobial functions independent of autophagic targeting of any kind (Canton et al., 2021; Herb and Schramm, 2021). Otherwise, when PINCA is induced in the presence of ROS, it is likely that also LAP is induced in parallel to PINCA, making it difficult to distinguish between these two pathways. The identification of a mechanistic component, which exclusively activates PINCA in the presence of functional ROS production, but without activating LAP, will be necessary to address this unanswered question.

Notably, in addition to enhanced phagolysosomal fusion, we found that LC3-positive phagosomes formed by PINCA were damaged less often than conventional, LC3-negative phagosomes (Gluschko et al., 2021). This indicates that either targeting by PINCA impedes the damage to the phagosomal membrane, or that LC3-decorated phagosomes are pre-assigned for membrane damage repair, as observed during autophagy induced in *S. typhimurium*-infected epithelial cells (Thurston et al., 2012; Kreibich et al., 2015). Thus, PINCA might represent an attempt of macrophages to repair damaged phagosomal membranes as last resort against the bacteria, which have not yet escaped from the phagosome.

## Antibacterial Xenophagy in Macrophages

Several bacteria manage to escape form the phagosome *via* rupture of the phagosomal membrane prior to degradation or targeting by LAP or PINCA (Fernandez-Prada et al., 2000; Hamon et al., 2012; Jamwal et al., 2016; Bell et al., 2021). Escape into the cytosol not only means the evasion from degradation, but provides also a rich pool of nutrients for the escaped pathogen, which enables cytosolic replication within the cell without being detected by other phagocytes. However, macrophages have established a counter measure to recapture cytosolic bacteria, namley xenophagy (Sharma et al., 2018) (**Figure 1**). During xenophagy, the cargo for the autophagic machinery is a bacterial pathogen that is escaping or has already escaped from the phagosome/vacuole (Pao and Rape, 2019). The cytosolic bacterium can be tagged with ubiquitin and recognized by various autophagy receptors, e.g. Sequestosome-1 (SQSTM1)/p62 or calcium-binding and coiled-coil domain-containing protein 2 (CALCOCO2)/NDP52 (Thurston et al., 2009; Johansen and Lamark, 2011), which recruit the autophagic components to the target. Thereby, the cargo is enclosed and isolated from the rest of the cell by formation of a double-membrane structure, called phagophore (Yla-Anttila et al., 2009; Lamb et al., 2013; Chang et al., 2021). Initiation of phagophore formation is, in contrast to LAP and PINCA, dependent on activation of the ULK complex (Suzuki et al., 2007; Jung et al., 2009; Koyama-Honda et al., 2013; Fujioka et al., 2014; Shi et al., 2020; Mercer et al., 2021), which is composed of ULK1 or ULK2 (ULK1/2), FIP200, ATG13, as well as ATG101 (Chang and Neufeld, 2009; Ganley et al., 2009; Hara and Mizushima, 2009; Hosokawa et al., 2009; Mercer et al., 2009; Lin and Hurley, 2016; Hurley and Young, 2017; Chang et al., 2021). ULK complex activity, which is not required for LAP (Martinez et al., 2011; Martinez et al., 2015) or PINCA (Gluschko et al., 2021), leads to the generation of PI3P at the membrane of the forming phagophore *via* one of the two PI3KC3 complexes (Russell et al., 2013; Backer, 2016). The two PI3KC3 complexes activated during macroautophagy contain the same core components, namely VPS34 and VPS15, BECN1 and AMBRA1 (Yu et al., 2015; Young et al., 2019), but can either recruit ATG14 (found in the PI3KC3 complex 1) or UVRAG (found in the PI3KC3 complex 2) (Itakura et al., 2008), (**Figure 1**). The PI3P generated at the membrane serves as a platform for recruitment of a complex consisting of WIPI proteins and ATG2, which are dispensable for LAP (Martinez et al., 2015; Fischer et al., 2020). After recruitment to the forming membrane of the phagophore, the WIPI-ATG2 complex itself recruits the LC3 conjugation machinery (Kabeya et al., 2000; Martens, 2016; Schaaf et al., 2016). Notably, ULK1 inhibits the catalytic activity of ATG4 by phosphorylation, thereby preventing the deconjugation of LC3 from the autophagosome (Pengo et al., 2017). In addition, cytosolic ROS can also inhibit ATG4 deconjugation activity (Scherz-Shouval et al., 2007), similar to Nox2-mediated oxidative inactivation of ATG4 during LAP (Ligeon et al., 2021). Finally, the closed autophagosome subsequently fuses with lysosomes, which leads to degradation of the recaptured bacterial pathogen in an autophagolysosome (Sharma et al., 2018). Xenophagy therefore plays a crucial role in the cellular defense against invading bacteria (Levine et al., 2011), not only in macrophages (Niu et al., 2008; English et al., 2009; Moreau et al., 2010; Travassos et al., 2010; Starr et al., 2012; Park et al., 2016; Ganesan et al., 2017; Zhou et al., 2017), but also in non-immune cells (Gutierrez et al., 2004; Birmingham et al., 2006; Thurston et al., 2009; Zheng et al., 2009; Wild et al., 2011; Thurston et al., 2012; von Muhlinen et al., 2012; Xu et al., 2019).

However, bacteria have also evolved mechanism to avoid degradation by xenophagy (Huang and Brumell, 2014). Some bacteria can reside in autophagosomes or autophagosome-like structures for replication, such as *H. pylori* (Wang et al., 2009; Hu et al., 2019), *Legionella pneumophila* (Amer and Swanson, 2005; Joshi and Swanson, 2011) or *Yersinia pseudotubercolosis* (Moreau et al., 2010), while others completely inhibit xenophagy and freely replicate in the cytosol. *L. monocytogenes*, for example, inhibits xenophagy *via* the virulence factors actin assembly-inducing protein (ActA) (Yoshikawa et al., 2009) and the two phosphatidylinositol-specific phospholipases C PlcA and PlcB (Mitchell et al., 2015), *S. flexneri* inhibits binding of ATG5 during xenophagy *via* the virulence factor IscB (Ogawa et al., 2005) and *S. typhimurium* secrets several virulence factors, such as SseL (Mesquita et al., 2012), SseF and SseG (Feng et al., 2018) to counter xenophagic targeting (Casanova, 2017).

## Conclusions

Macrophages are among the first line of defense against invading pathogens. Due to distinct virulence factors, some bacterial pathogens can evade the destruction in the phagosome, either by re-modulation of the phagosomal milieu or *via* escape into the cytosol. Activation of LAP, a highly microbicidal non-canonical autophagy pathway (Herb et al., 2020; Grijmans et al., 2022), enhances the degradative capacity of macrophages. We recently described another non-canonical autophagy pathway termed PINCA (Gluschko et al., 2021), which is triggered by perforation of bacteria-containing phagosomes, independent of the ULK complex components ULK1/2 and FIP200 and also independent of Nox2-derived ROS, therefore representing a non-canonical autophagy pathway distinct from LAP. Pore-forming toxins can induce osmotic imbalances, which are sensed by V-ATPase and result in ATG16L recruitment and LC3 conjugation (Florey et al., 2015). It is very likely that LC3 recruitment to perforated phagosomes during PINCA is also activated by the V-ATPase-ATG16L1-axis, which might represent a general pathway to recruit LC3 to damaged, yet not ruptured compartments. Furthermore, it is reasonable that also during LAP, V-ATPase is responsible for ATG16L1 recruitment and subsequent LC3 conjugation, since ROS production by Nox2 is not sufficient to induce LAP, when V-ATPase is inhibited (Hooper et al., 2021). It is tempting to speculate that LC3 conjugation during LAP is not triggered by Nox2-generated ROS but by V-ATPase-induced ATG16L1 recruitment. Instead, ROS production during LAP only prevents deconjugation of LC3 through oxidative inactivation of ATG4 as shown by Ligeon et al. (2021), which resembles redox-dependent inactivation of ATG4 during autophagy (Scherz-Shouval et al., 2007; Pérez-Pérez et al., 2016). While LAP and xenophagy have clear degradative functions, despite some bacterial pathogens exploiting autophagosomes as a replicative niche (Huang and Brumell, 2014; Siqueira et al., 2018; Riebisch et al., 2021), the functional purpose of PINCA remains unclear. It is possible that PINCA might represent an emergency repair mechanism for damaged phagosomes, similar to membrane repair mechanisms in *S. typhimurium*-infected epithelial cells (Thurston et al., 2012; Kreibich et al., 2015). Alternatively, LC3 on the perforated phagosome may recruit an entire spectrum of proteins containing a LC3-interacting region (Johansen and Lamark, 2020), which in turn may accelerate phagolysosomal fusion, or exert another, yet unknown function of PINCA.

## Data Availability Statement

The original contributions presented in the study are included in the article/supplementary material, further inquiries can be directed to the corresponding author.

## Author Contributions

Conceptualization, MH. writing—original draft preparation, MH, AG and MK. writing—review and editing, MH, AG, AF and MK. visualization, MH. supervision, MK. project administration, MK. funding acquisition, MH, AG and MK. All authors have read and agreed to the published version of the manuscript.

## Funding

This work was supported by the Deutsche Forschungsgemeinschaft (DFG) grant SCHR 1627/2-1 and intramural grants to A.G. and M.H and by the Federal state grant (NRW) 323-8.04.10.02-141905 to MK.

## Conflict of Interest

The authors declare that the research was conducted in the absence of any commercial or financial relationships that could be construed as a potential conflict of interest.

## Publisher’s Note

All claims expressed in this article are solely those of the authors and do not necessarily represent those of their affiliated organizations, or those of the publisher, the editors and the reviewers. Any product that may be evaluated in this article, or claim that may be made by its manufacturer, is not guaranteed or endorsed by the publisher.
